# Association Between Arch-Shaped Hypo-Autofluorescent Lesions Detected Using Fundus Autofluorescence and Postoperative Hypotony

**DOI:** 10.3390/jcm13206264

**Published:** 2024-10-20

**Authors:** Yuji Yoshikawa, Jun Takeuchi, Aya Takahashi, Masaharu Mizuno, Tomoka Ishida, Takashi Koto, Makoto Inoue

**Affiliations:** 1 Kyorin Eye Center, Kyorin University School of Medicine, 6-20-2 Shinkawa, Mitaka, Tokyo 186-8611, Japan; yuji.yoshi.md@gmail.com (Y.Y.); jun-takeuchi@ks.kyorin-u.ac.jp (J.T.); ayatak1126@gmail.com (A.T.); m-mizuno@ks.kyorin-u.ac.jp (M.M.); tom-oph@ks.kyorin-u.ac.jp (T.I.); koto@ks.kyorin-u.ac.jp (T.K.); 2 Department of Ophthalmology, Saitama Medical University, 38 Morohongo, Saitama 350-0495, Japan

**Keywords:** chorioretinal fold, fundus autofluorescence, microincision vitrectomy surgery, postoperative hypotony, vitrectomy

## Abstract

**Background:** Chorioretinal folds are observed after vitrectomy due to ocular collapse caused by low intraocular pressure. The purpose of this study is to investigate the relationship between the postoperative hypotony, chorioretinal folds, and the fundus autofluorescence (FAF) findings. **Methods:** Two-hundred-and-seventy consecutive eyes that had undergone 25- or 27-gauge vitrectomy were examined. The associations between the arch-shaped hypo-autofluorescent lesions in the FAF images and the postoperative hypotony with intraocular pressure (IOP) ≤ 4 mmHg were determined on the day after the surgery. **Results:** Arch-shaped hypo-autofluorescent lesions were seen in 4 of the 270 eyes (1.5%), and hypo-autofluorescence was observed in 3 of 14 hypotonic eyes (18.5%). This was significantly more frequent than in the non-hypotony group (0.4%, *p* = 0.0004). Optical coherence tomography showed a loss of the ellipsoid zone and retinal pigment epithelial layer in the region of the arch-shaped lesions. None of the arch-shaped hypo-autofluorescent lesions involved the fovea, and the vision recovered in all cases. The hypo-autofluorescent lesions did not disappear during the 4 to 16 month observation period. **Conclusions:** The postoperative arch-shaped hypo-autofluorescent lesions were associated with postoperative hypotony and RPE damage due to chorioretinal folds. These findings remained even when the IOP was normalized and chorioretinal folds disappeared.

## 1. Introduction

Pars plana vitrectomy is performed for various retinal diseases, and in recent years, microincision and minimally invasive techniques have been used. Microincision vitrectomy surgery (MIVS) with 25-gauge (G) and 27G instruments have been performed with greater safety and reduced complications [1,2,3]. However, postoperative hypotony is one of the complications after vitrectomy [4,5,6,7,8,9,10,11,12,13,14,15,16]. The presence of early postoperative hypotony after 25G pars plana vitrectomy with nonexpansive endotamponade was highly associated with a younger age, prior vitreoretinal surgeries, higher incidence of preoperative ocular hypertension, pseudophakia, silicone oil removal, and use of external diathermy [15]. Bourgault and associates [12] compared the incidence of hypotony after oblique and straight sclerotomies in 25G transconjunctival sutureless vitrectomy. They reported that the incidence of hypotony was not significant different between eyes which had oblique and straight incisions. It has also been reported that the incidence of hypotony was significantly lower in eyes with air or gas tamponade than in fluid-filled eyes [12]. The incidence of ciliochoroidal detachment at sclerotomy sites was detected by anterior segment swept-source optical coherence tomography in 63.3% of 30 eyes with an epiretinal membrane that underwent MIVS during a 1-day observation period [13]. The mean postoperative intraocular pressure (IOP) was significantly lower than the preoperative IOP in eyes with a ciliochoroidal detachment. The mean postoperative IOP was significantly higher in eyes without a ciliochoroidal detachment than in eyes with a ciliochoroidal detachment. The incidence of open sclerotomies was significantly higher in eyes with a ciliochoroidal detachment than in eyes without a ciliochoroidal detachment at 3 h postoperation. Hypotony during the early postoperative period appeared to be associated with a ciliochoroidal detachment at the sclerotomy sites [13].

Earlier studies have shown that in hypotonic eyes with choroidal folds, the retinal pigment epithelium (RPE) is stretched at the crest of the fold and compressed at the trough. These morphological alterations resulted in hyperfluorescence at the crest and hypofluorescence at the trough in the fluorescein angiograms (FA). There is also hyper-autofluorescence at the crest and hypo-autofluorescence at the trough of the fundus autofluorescence (FAF) images [17,18].

We have had cases of postoperative hypotony after vitrectomy that developed arch-shaped hypo-autofluorescent lesions that were coincident with the choroidal folds. However, the relationship between the hypotony and the FAF images has not been determined.

Thus, the purpose of this study was to determine the relationship between the postoperative hypotony and the arch-shaped hypo-autofluorescent lesions that developed after routine pars plana vitrectomy.

## 2. Materials and Methods

This single-centre observational study was approved by the Institutional Review Committee of the Kyorin University School of Medicine (R04-056). It adhered to the tenets of the Declaration of Helsinki. All of the patients received a detailed explanation of the surgical and ophthalmic procedures, and all signed an informed consent form. All of the patients consented to our review of their medical records and their anonymized use in medical publications.

### 2.1. Subjects

We reviewed the medical records of 270 consecutive eyes that had undergone routine 25G and 27G pars plana vitrectomy (PPV) between January 2021 and February 2022 and had FAF examinations. The patients were followed for at least 3 months. The procedures used for the vitrectomy were approved by the Kyorin University Hospital Ethics Committee (Reference number; R04-056), and they conformed to the tenets of the Declaration of Helsinki.

### 2.2. Measurement of Chorieretinal Folds by Optical Coherence Tomography (OCT) and Fundus Autofluorescence (FAF)

The eyes on which a single surgeon (M.I.) had performed the vitrectomy were studied. We collected the age, sex, pre- and postoperative best-corrected visual acuity (BCVA), pre- and postoperative IOP, and axial length from the medical records. OCT images (Spectralis, Heidelberg Engineering, Heidelberg, Germany), infrared scanning laser ophthalmoscopic (SLO) images (Spectralis, Heidelberg Engineering, Heidelberg, Germany), and widefield FAF images (200Tx^®^ or California, Optos Inc., Dunfermline, UK) were also collected. The frequency of arch-shaped hypo-autofluorescent lesions in the FAF images before and after vitrectomy was determined. Then, we determined the association between the arch-shaped hypo-autofluorescent lesions and the pre- and postoperative findings.

### 2.3. Statistical Analyses

All data are expressed as the means ± standard deviations. A postoperative hypotony was defined as an IOP of ≤4 mmHg on the day after the surgery. Non-parametric tests (Fischer’s exact test and Mann–Whitney *U* test) were used to determine the significance of the differences in the systemic and ocular parameters between the hypotony and non-hypotony groups. All statistical analyses were performed using SPSS (version 28.0; IBM, Armonk, New York, NY, USA).

## 3. Results

In total, 270 eyes from 270 patients (120 women; 150 men) met the inclusion criteria. The mean age of all participants was 64 ± 14 (±standard deviation) years, and the mean axial length was 25.2 ± 2.1 mm. The pre- and postoperative BCVA was 0.55 ± 0.67 and 0.16 ± 0.47 logarithm of the minimum angle of resolution (logMAR) units, respectively. The pre- and postoperative IOP was 14.6 ± 4.3 and 13.2 ± 6.0 mmHg, respectively. In total, 77 eyes (29%) underwent 25G vitrectomy, and 193 eyes (71%) underwent 27G vitrectomy. Of the 270 eyes, 73 eyes (27%) had an epiretinal membrane, 59 eyes (22%) had a macular hole, 22 eyes (8%) had a dislocation of intraocular lens, 51 eyes (19%) had a rhegmatogenous retinal detachment and/or proliferative vitreoretinopathy, and 28 eyes (10%) had proliferative diabetic retinopathy (Table 1).

**Figure 1:**
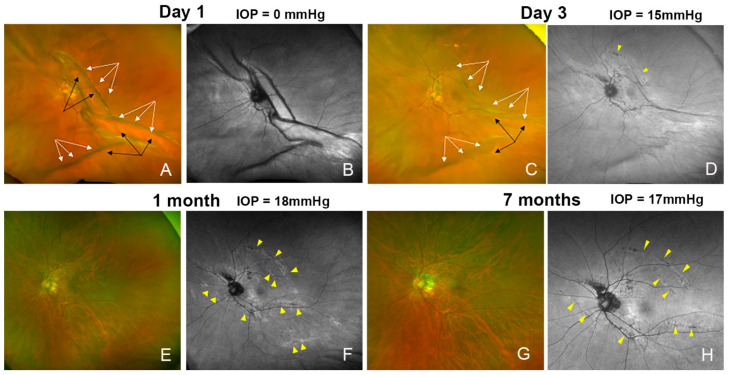
Postoperative fundus photograph and fundus autofluorescent (FAF) images of an eye with an epiretinal membrane in a 64-year-old woman. (**A**) Fundus photograph showing that the eye appears to be deformed by the very low intraocular pressure (IOP) on the day after the surgery, resulting in deformation and severe chorioretinal infoldings with crests (black arrows) and troughs (white arrows). (**B**) FAF image showing the chorioretinal infoldings next to the shadowed area. (**C**) On day 3, the chorioretinal folds are reduced and the IOP is normal. The crests (black arrows) and troughs (white arrows) are flattened. (**D**) FAF image shows arch-shaped hypo-autofluorescent lesions (yellow arrowheads) corresponding to the troughs of the chorioretinal folds with a dark shadowed area next to it. (**E**) At one month, the chorioretinal folds are not present, and vision has recovered to 20/20. (**F**) FAF image shows the arch-shaped hypo-autofluorescent lesions (yellow arrowhead) with hyper-autofluorescent lesions near the hypo-autofluorescent lesions. (**G**,**H**) The hypo-autofluorescent lesions (yellow arrowhead) persisted at 7 months, and were present for 12 months postoperatively.

**Figure 2:**
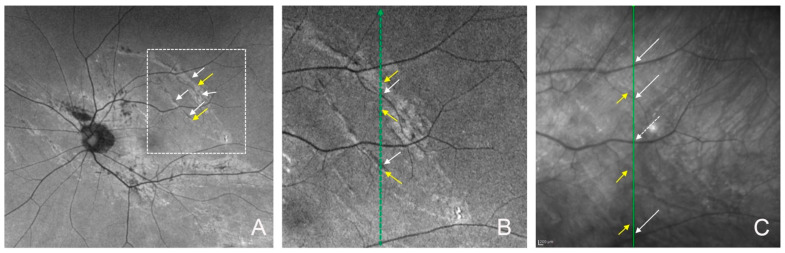

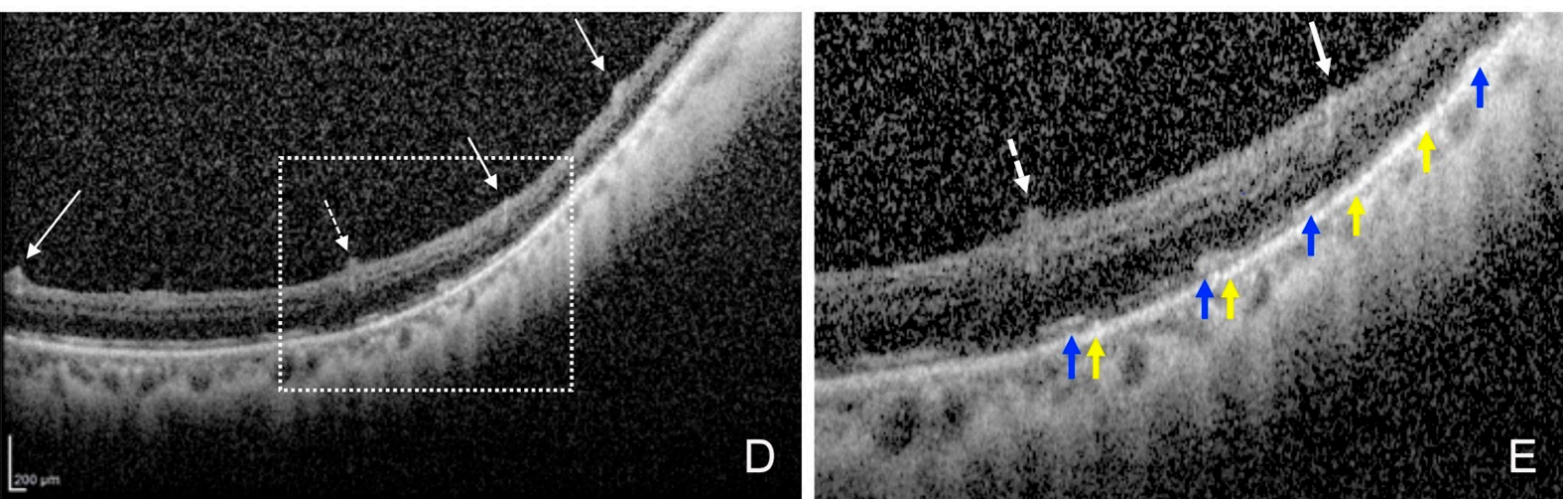
Postoperative fundus autofluorescence (FAF) and optical coherence tomography (OCT) images of the upper chorioretinal fold of the same eye as Figure 1 at one month. (**A**) FAF image and (**B**) magnified FAF image (the dashed square of **A**) show the arch-shaped hypo-autofluorescent lesions (white arrows) with hyper-autofluorescent lesions (yellow arrows). (**C**) Infrared image showing arch-shaped bright lesions (yellow arrows) corresponding to the hypo-autofluorescent lesions in the FAF image. White arrows point to a retinal artery, white dots arrow point to a retinal vein. (**D**) OCT image and (**E**) magnified OCT image (the dashed square of **D**) of a vertical scan (green line of Figure 2B,C) showing the hyperreflective area of the retinal pigment epithelium (yellow arrows), hyperreflective area of the photoreceptor outer segments (blue arrows), and disruption of the ellipsoid zone. White arrows: retinal artery; white dotted arrow: retinal vein.

**Figure 3:**
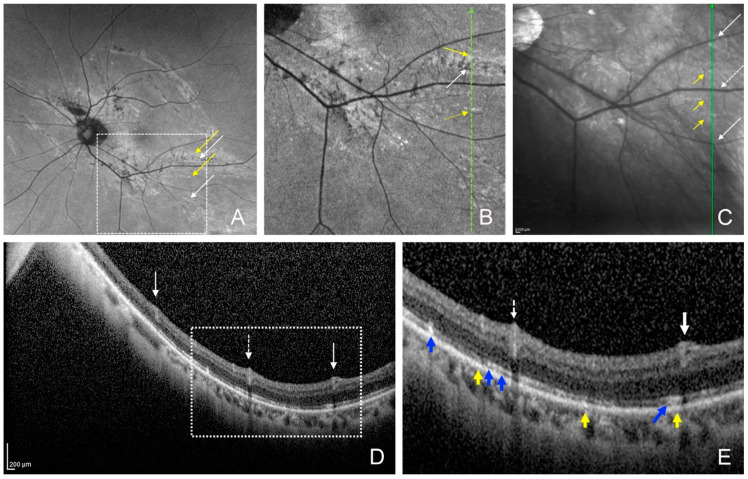
Postoperative fundus autofluorescence (FAF) and optical coherence tomographic (OCT) images of the lower chorioretinal fold of the same eye as Figure 1 at one month. (**A**) FAF image and (**B**) magnified FAF image (the dashed square of **A**) showing the arch-shaped hypo-autofluorescent lesions (white arrows) with hyper-autofluorescent lesions (yellow arrows). (**C**) Infrared image showing bright lesions (yellow arrows) corresponding to the hypo-autofluorescent and hyper-autofluorescent lesions in the FAF image. White arrows point to retinal artery, white dots arrows point to retinal vein. (**D**) OCT image and (**E**) magnified OCT image (the dashed square of **D**) of a vertical scan (green line in Figure 3B,C) showing the hyperreflective area of retinal pigment epithelium and photoreceptor outer segments (yellow arrows), the hyperreflective area of ellipsoid zone band (blue arrows), and disruption of the ellipsoid zone. White arrows: retinal artery; white dotted arrow: retinal vein.

**Figure 4:**
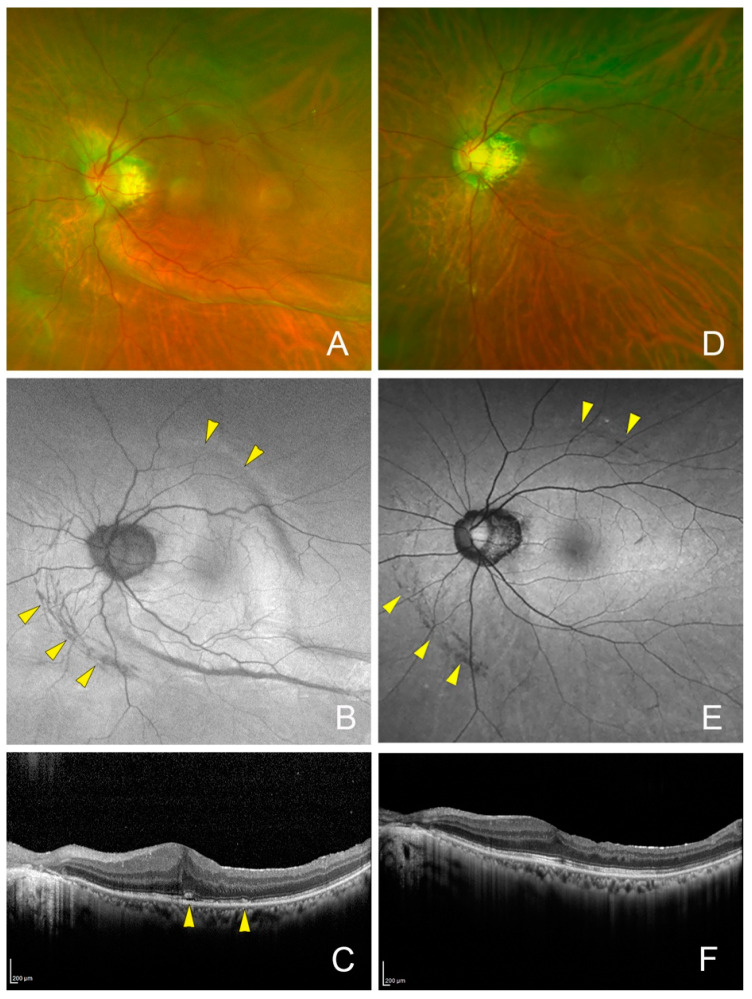
Postoperative fundus photograph and fundus autofluorescent (FAF) images of the left eye of a 63-year-old man with an epiretinal membrane after treatment for a retinal detachment. (**A**) Fundus photograph showing that the eye appears to be deformed by a low intraocular pressure (IOP) of 2 mmHg on day 1 after the surgery. (**B**) Postoperative FAF image on postoperative day 1 showing arched hypo-autofluorescent lesions (yellow arrowheads). (**C**) Optical coherence tomographic (OCT) image of a horizontal section on postoperative 6 days showing the hyperreflective area of the photoreceptor outer segments (yellow arrowheads). (**D**) Postoperative image and FAF image (**E**) at 7 months showing no chorioretinal folds, but persistent arched hypo-autofluorescent lesions (yellow arrowheads). Vision improved to 20/18. (**F**) OCT image showing an absence of the hyperreflective area of the photoreceptor outer segments.

The arch-shaped hypo-autofluorescent findings were seen in 4 of the 270 eyes (1.5%) and in 3 of 14 hypotonic eyes (18.5%), which was significantly more frequent than in the non-hypotony eyes (0.4%, *p* = 0.0004, Table 2 and Table 3). In three eyes with markedly low IOP on the day after the surgery, chorioretinal infoldings were observed due to an ocular collapse (Figure 1, Figure 2, Figure 3 and Figure 4). The FAF images also showed chorioretinal infoldings with the shadowed area corresponding to the trough (valley) of the chorioretinal infoldings. Intravitreal air injection was performed immediately to treat the low IOP. The IOP improved 3 days after the surgery, and the choroidal folds were also improved in all three eyes. The FAF images showed arch-shaped hypo-autofluorescent lesions, which corresponded to the troughs of the chorioretinal folds next to the dark shadowed area.

One month after the surgery, fundus examination showed that the chorioretinal folds were not present, but the FAF images showed that the arch-shaped hypo-autofluorescent lesions with hyper-autofluorescent lesions near the hypo-autofluorescent lesions were still present (Figure 1, Figure 2 and Figure 3). The infrared scanning laser ophthalmoscopic (SLO) images showed that the arch-shaped bright lesions corresponded to the hypo-autofluorescent lesions in the FAF images. The OCT images showed a hyperreflective area of the retinal pigment epithelium and hyperreflective area of photoreceptor outer segments next to the area of the disrupted ellipsoid zone band. The Humphrey 30-2 visual field test did not detect any decreased sensitivity at the site corresponding to the chorioretinal folds in two eyes.

**Figure 5:**
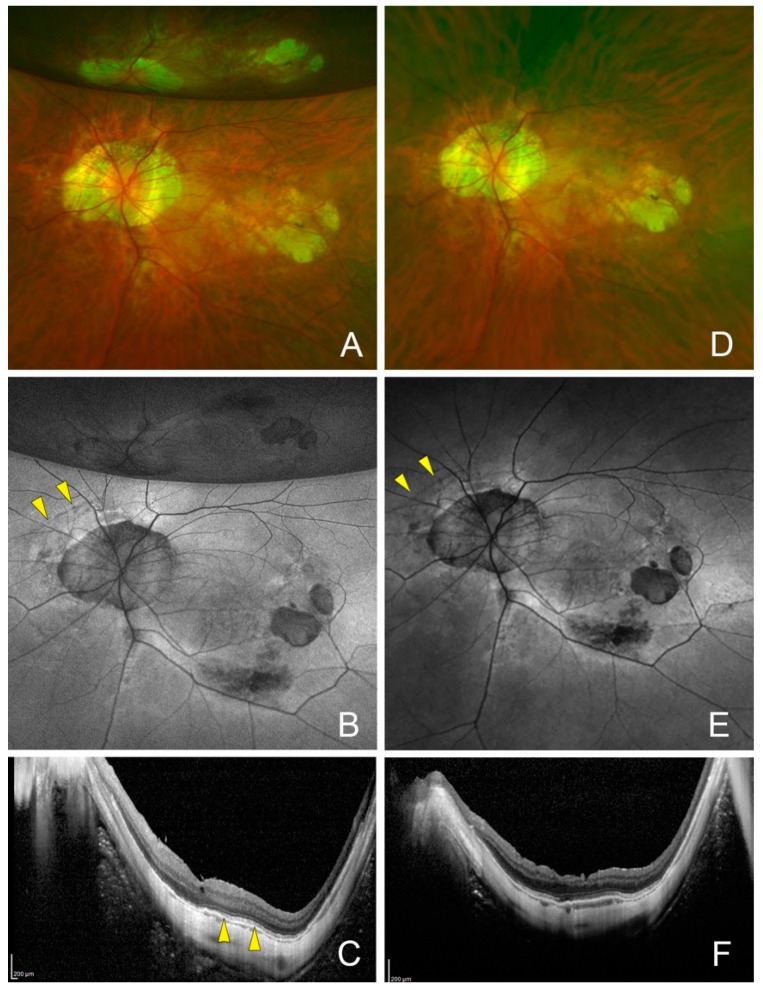
Postoperative fundus photograph and fundus autofluorescent (FAF) images of the left eye of a 64-year-old man with a lamellar macular hole. (**A**) Fundus photograph on postoperative day 7 showing that the eye has recovered from the deformity present on the day after the surgery. Intraocular pressure (IOP) has recovered to 16 mmHg from 3 mmHg on day 1. Intravitreal tamponade air is still present. (**B**) Postoperative FAF image shows arched hypo-autofluorescent lesions (yellow arrowheads) on the nasal side. (**C**) Optical coherence tomographic (OCT) images in a horizontal section on postoperative day 7 showing the hyperreflective elongated area of the photoreceptor outer segment layer (yellow arrowheads). (**D**) Postoperative images and (**E**) FAF image at 4 months after vitrectomy shows the persisted arched hypo-autofluorescent lesions (yellow arrowheads). Vision has improved to 20/20. (**F**) OCT image showing the absence of the hyperreflective area of the photoreceptor outer segment layer.

**Figure 6:**
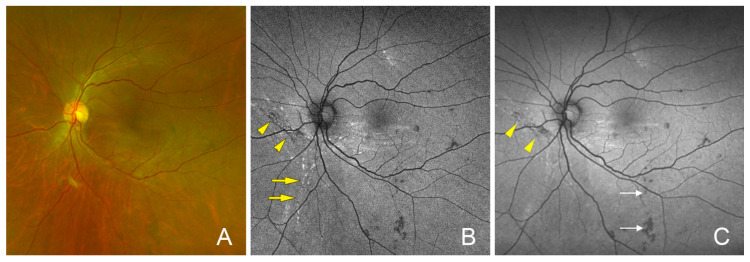
Postoperative images and fundus autofluorescent (FAF) images of the left eye of a 55-year-old man after vitrectomy for a dislocated intraocular lens. (**A**) Fundus photograph on postoperative one month showing no chorioretinal folds. IOP on postoperative day 1 was 12 mmHg. (**B**) FAF image showing arched hypo-autofluorescent lesions (yellow arrowheads) and hyper-autofluorescent lesions (yellow arrows) near and away from the hypo-autofluorescent lesions. (**C**) FAF images at postoperative 7 months showing the persistent hypo-autofluorescent lesions (yellow arrowheads). The hyper-autofluorescent lesions have become fuzzy. Vision was 20/133 due to a previous retinal detachment. Hypo-autofluorescent dots (arrows) were seen before the surgery.

Arched hypo-autofluorescent lesions in the FAF images were observed nasal to the optic disc in all four eyes, and the lesion did not extend to the macula in all four eyes (Figure 1, Figure 2, Figure 3, Figure 4, Figure 5 and Figure 6). These findings were observed until the last postoperative examination at 16 months. The postoperative BCVA did not differ significantly between the eyes with and without the hypo-autofluorescent lesions (Table 2).

## 4. Discussion

The arch-shaped hypo-autofluorescent lesions seen in the FAF images after vitrectomy were seen significantly more frequently in the postoperative hypotony group. The sites of these lesions corresponded with the location of the chorioretinal folds, which suggests that they were related to the ocular collapse caused by postoperative hypotony. In addition, these findings persisted for up to 12 months after the surgery, even when the IOP was normalized. The axial length of the eyes in the postoperative hypotony group was significantly longer than that of the non-hypotony group, which indicates that the recovery from early postoperative hypotony was affected by the size of the eyeball. However, the incidence of arch-shaped hypo-autofluorescent lesions was not related to the axial length, but rather to the ocular collapse caused by postoperative hypotony.

In general, hypotony is known to occur after trauma or glaucoma filtering surgery [19,20]. In cases with chorioretinal folds caused by hypotonic maculopathy, the RPE cells are compressed together in the troughs of the folds, resulting in the hyper-autofluorescent lesions in the FAF images. The RPE atrophy occurs at the crests of the folds. These changes result in the hypo-autofluorescent lesions in the FAF images. On the other hand, a posterior staphyloma in highly myopic eyes is known to cause hypo-autofluorescent lesions due to RPE atrophy caused by the expansion of the eyeball [21].

The arched hypo-autofluorescent lesions with hyper-autofluorescent lesions in the FAF images were detected at the troughs of the chorioretinal folds. These folds represent RPE damages due to deformation of the eyeball. In contrast, the hypo-autofluorescent or hyper-autofluorescent lesions in the FAF images were not detected at the crests of the chorioretinal folds. The OCT images revealed hyperreflective areas of the RPE and the photoreceptor outer segments, which represented aggregated RPE cells and the accumulation of photoreceptor outer segments due to RPE damage. The region of the ellipsoid zone in the OCT images also corresponded to the hypo-autofluorescent lesion caused by the RPE damage. The infrared SLO images showed that the arch-shaped bright lesions also corresponded to the RPE damage by their increased reflectivity. These findings in the FAF and SLO images may differ because the chorioretinal folds were displaced and recovered after the recovery from hypotony.

The RPE atrophy was not clearly seen in the OCT images, but the damage of the ellipsoid zone and RPE in the troughs of the chorioretinal fold caused by the extraocular deformation appeared to be more severe than the deformation that usually occurs in hypotonic maculopathy. These changes resulted in the hypo-autofluorescent lesions in the FAF images. There were also cases of arched hyper-autofluorescent lesions located next to hypo-autofluorescent lesions. Hypotonic maculopathy typically occurs after glaucoma surgery. However, the changes in our study were different from the changes seen in hypotonic maculopathy after glaucoma surgery. Possibly, the absence of the vitreous gel made it difficult to maintain the ocular morphology at low IOP, resulting in a more severe ocular collapse and chorioretinal folds that can damage the RPE and photoreceptor cells. Experiments in animal models of myopia have reported that stretching the RPE causes cell expansion and proliferation [22]. The degree of scleral deformation was less at sites away from the chorioretinal troughs, suggesting that the hyper-autofluorescent lesions were caused by RPE aggregation, as seen in hypotonic maculopathy.

These findings did not occur in the macular area and did not affect the postoperative visual acuity. The severe postoperative hypotony on the first postoperative day was treated immediately by the intravitreal injection of air. However, the absence of deformation might be because the location of the optic disc prevented the deformation of the macular area. The chorioretinal folds were described to radiate outward temporally in a branching fashion from the optic disc. However, nasal to the disc, the folds tended to be arranged concentrically by the traction exerted nasally by the optic nerve fibres as they moved [23,24]. One of the four eyes with the postoperative arch-shaped hypo-autofluorescent lesions did not have low IOP the day after the surgery, and no retinal choroidal folds were seen on fundus examination on the next day. The reason for this is not clear, but it is possible that the eye had low IOP immediately after surgery and the IOP had recovered on the next day.

Our study has several limitations. First, the very small number of eyes studied and the retrospective nature limited the strength of the conclusions that can be made. Second, the number of the patients with choroidal fold was too few to perform statistical analyses of findings of the FAF and OCT images. Third, the follow-up period may be too short to make a conclusion to evaluate the eventual outcome of hyper-autofluorescent and hypo-autofluorescent lesions.

## 5. Conclusions

In conclusion, postoperative arch-shaped hypo-autofluorescent lesions were associated with the postoperative hypotony and RPE damage due to the chorioretinal folds. These changes remained even after the IOP recovered and the chorioretinal folds disappeared.

## Figures and Tables

**Table 1:** Demographics of the patients.

Age (years)	64 ± 14
Sex (woman/man)	120/150
Preoperative BCVA (logMAR units)	0.55 ± 0.67
Postoperative BCVA (logMAR units)	0.16 ± 0.47
Preoperative IOP (mmHg)	14.6 ± 4.3
Postoperative IOP (mmHg)	13.2 ± 6.0
Axial length (mm)	25.2 ± 2.1
Arch-shaped hypo-autofluorescent lesions (eyes [%])	4 eyes (1.5%)
Gauge of the vitrectomy (eyes [%])	25G: 77 (29%) 27G:193 (71%)
Pathologies (eyes [%])	
ERM	73 (27%)
MH	59 (22%)
IOL dislocation	22 (8%)
RRD/PVR	51 (19%)
PDR	28 (10%)
Other	37 (14%)

BCVA = best-corrected visual acuity, IOP = intraocular pressure, ERM = epiretinal membrane, MH = macular hole, IOL = intraocular lens, RRD = rhegmtogenous retinal detachment, PVR = proliferative vitreoretinopathy, PDR = proliferative diabetic retinopathy.

**Table 2:** Comparison of cases with or without arch-shaped hypo-autofluorescent lesions.

	Arch-Shaped Hypo-Autofluorescent Lesions (+)	(−)	*p*-Value
Eyes	4	266	-
Age, years	61 ± 5	64 ± 14	0.573 *
Preoperative BCVA (logMAR units)	0.06 ± 0.05	0.56 ± 0.68	0.050 *
Postoperative Maximum BCVA (log MAR units)	0.19 ± 0.43	0.16 ± 0.47	0.752 *
Pre-IOP (mmHg)	14.3 ± 2.2	14.6 ± 4.3	0.948 *
Axial length (mm)	27.7 ± 3.5	25.1 ± 2.0	0.114 *
Postoperative Hypotony (+)	3 (18.5%)	13 (4.8%)	0.0004 †
Postoperative Hypotony (−)	1 (0.4%)	253 (93.7%)	-

BCVA = best-corrected visual acuity, IOP = intraocular pressure. * Mann–Whitney *U* test, † Fischer’s exact test.

**Table 3:** Comparison of cases with or without postoperative hypotony.

	Hypotony Group, (n = 16, 5.9%)	Non-Hypotony Group, (n = 254, 94.1%)	*p*-Value
Age, year	62 ± 11	64 ± 14	0.387 *
Preoperative BCVA (logMAR)	0.33 ± 0.51	0.57 ± 0.68	0.139 *
Postoperative Maximum BCVA (log MAR)	0.11 ± 0.29	0.17 ± 0.48	0.431 *
Pre-IOP (mmHg)	14.6 ± 3.0	14.6 ± 4.3	0.874 *
Axial length (mm)	26.7 ± 2.7	25.2 ± 2.1	0.011 *
Arch-shaped hypo-autofluorescent lesions, eyes (%)	3 (18.5%)	1 (0.4%)	0.0004 †
Gauge of the vitrectomy (eyes [%])	25G: 2 (12.5%),	25G: 75 (29.5%),	0.112 †
	27G: 14 (87.5%)	27G: 179 (70.5%)	
Pathologies (eyes [%])			
ERM	6 (38%)	67 (26%)	
MH	3 (19%)	56 (22%)	
IOL/lens dislocation	5 (31%)	17 (7%)	
RRD/PVR	0 (0%)	51 (20%)	
PDR	1 (6%)	27 (11%)	
others	1 (6%)	36 (14%)	

BCVA = best-corrected visual acuity, IOP = intraocular pressure, ERM = epiretinal membrane, MH = macular hole, IOL = intraocular lens, RRD = rhegmtogenous retinal detachment, PVR = proliferative vitreoretinopathy, PDR = proliferative diabetic retinopathy. * Mann–Whitney *U* test, † Fischer’s exact test.

## Data Availability

The data presented in this study are available on request from the corresponding author (M.I.).
